# Climatic and environmental aspects of the Mongol withdrawal from Hungary in 1242 CE

**DOI:** 10.1038/srep25606

**Published:** 2016-05-26

**Authors:** Ulf Büntgen, Nicola Di Cosmo

**Affiliations:** 1 Swiss Federal Research Institute WSL, Zürcherstrasse 111, 8903 Birmensdorf, Switzerland; 2 Oeschger Centre for Climate Change Research, Falkenplatz 16, 3012 Bern, Switzerland; 3 Global Change Research Centre AS CR, Zemedelska 1, 613 00 Brno, Czech Republic; 4 Institute for Advanced Study, School of Historical Studies, 1 Einstein Dr, Princeton, NJ 08540, USA; 5 Princeton University, Department of East Asian Studies, Princeton, NJ 08540, USA

## Abstract

The Mongol invasion of Eastern Europe, and especially its sudden withdrawal from Hungary in 1242 CE, has generated much speculation and an array of controversial theories. None of them, however, considered multifaceted environmental drivers and the coupled analysis of historical reports and natural archives. Here we investigate annually resolved, absolutely dated and spatially explicit paleoclimatic evidence between 1230 and 1250 CE. Documentary sources and tree-ring chronologies reveal warm and dry summers from 1238–1241, followed by cold and wet conditions in early-1242. Marshy terrain across the Hungarian plain most likely reduced pastureland and decreased mobility, as well as the military effectiveness of the Mongol cavalry, while despoliation and depopulation ostensibly contributed to widespread famine. These circumstances arguably contributed to the determination of the Mongols to abandon Hungary and return to Russia. While overcoming deterministic and reductionist arguments, our ‘environmental hypothesis’ demonstrates the importance of minor climatic fluctuations on major historical events.

The Mongols began their expansion in the early-13th century CE, and by 1279 had conquered much of Eurasia including China, central Asia, Russia and Iran (Fig. 1). Chinggis (Genghis) Khan (1162−1227), after being recognized as the sovereign of all Mongol peoples in 1206, led a series of victorious campaigns against the Jin dynasty in northern China, the Tangut state in northeast China and the Muslim kingdom of the Khwarezm in central Asia. A shift from slightly dryer to wetter climatic conditions in Mongolia after 1206 may have increased steppe productivity and thus have played a probable role in the buildup of Mongol power^1^,^2^. Chinggis Khan’s death in 1227 caused a brief lull in the conquest but under the reign of his son and successor Ögödei Khan (r. 1229–1241), new campaigns were launched, first of all completing the conquest of north China^3^.

Based on intelligence gathered in the course of military operation in central Asia, the Mongols turned west and attacked Russia in 1235, moving westwards across the Eurasian grassland belt. Under Batu, grandson of Chinggis Khan, they reached Ryazan in December 1237 (~200 km southeast of Moscow), and then proceeded to attack several other Russian towns, establishing their rule over Russia, which was going to be known as the Golden Horde^4^. The resistance of the joint Russian and Cuman (Turkic nomads) armies proved futile, and with the capture of Kiev in December 1240 the Mongols continued to invade much of the eastern parts of Europe.

Hungary is the westernmost extension of the steppe belt that forms a nearly uninterrupted grassland ‘highway’ reaching out from Mongolia. Batu and other notable Mongol commanders such as Qadan, Orda, Baydar, and Sübetei, together with probably 130,000 soldiers^5^, quickly proceeded to invade Hungary by crossing the Carpathian arc from multiple routes (Fig. 1), including the Verecke Pass and the Radna Pass in Romania. In the spring of 1241, showing an ability to coordinate military operations utterly unknown in Europe, the Mongols defeated Polish and Hungarian armies in two almost contemporaneous battles, at Leignitz in Poland on April 9 and at Mohi in Hungary on April 11 (Fig. 1). Following these victories, the Mongol armies regrouped in Hungary, and continued to occupy and devastate the region to the east of the Danube. The king of Hungary Béla IV fled to Austria and a Mongol army pursued him to the Dalmatian coast^6^. In 1241, the Mongols set up a preliminary administrative structure, possibly intending to prepare for long-term operation and occupation^7^. In early-1242, the Mongols crossed the Danube into western Hungary and, after a short two months of further devastation, suddenly began to withdraw via a southern route through Serbia and Bulgaria (Fig. 1), and from there back to Russia.

No reason is given in the Mongol sources to explain their abrupt departure from Hungary. Following a note in the report made by a papal legate, Friar Giovanni da Pian del Carpine, who visited the court of Güyük Khan in 1246 (successor of Ögödei, r. 1246–1248), many historians for a long time believed, that the Mongol withdrawal (MW) was due to the death of Great Khan Ögödei in December 1241 CE^8^. The ensuing political upheaval would have forced Batu to halt operations and return to Karakorum, the capital of the empire in Mongolia, to participate in political meetings to elect the new khan. This hypothesis, however, has been challenged on grounds that Batu never went back to Mongolia, and instead remained in the southern Russian steppes where he consolidated his power as ruler of the Golden Horde^9^.

Seeking more persuasive explanations, several other hypotheses have been introduced. One of them focuses on the actual purpose of the invasion, which was not to occupy the land but to punish the pastoral Cuman nomads, hostile to the Mongols, who had fled from the Volga region and sought refuge in Hungary^10^. The Mongols also sent threatening letters to the king of Hungary who had defied their power by hosting their enemies and, allegedly, harming their envoys^11^. Once the punishment had been administered, the Mongols had no reasons to stay.

Other concepts stressed the military difficulties met by the Mongols in storming fortified towns and also speculated that they had suffered significant losses that eventually made it difficult for them to continue the conquest^12^. One idea that, while critiqued, received broad attention, is centered on the carrying capacity of the Hungarian plain. This steppe region was insufficient to support the huge number of horses in Mongol army, which required ample pasture^9^. None of these concepts – political, military and environmental – is fully satisfactory, and each has undergone extensive scrutiny and critique^12^,^13^,^14^. At the same time, a combined multi-proxy assessment of documentary and paleoclimatic evidence is missing. A key argument for better understanding the climatological, geographical and ecological conditions of Hungary during the MW is that the environment was highly important in the decision-making process of the Mongol leadership, as shown in a letter by Hülegü to Louis IX of 1260 CE^15^.

Benefiting from recent work in paleoclimatic research^16^,^17^,^18^,^19^, we reanalyze high-resolution proxy archives from Europe and the last eight centuries. In order to recognize possible effects of past climate variability on human history, we first disentangle the mechanisms by which climate may have influenced both the unprecedented invasion as well as the rapid withdrawal of the Mongol troopers. Written historical sources^17^, in tandem with novel tree-ring and multi-proxy-based spatial field reconstructions of summer climate^18^,^19^, provide a geographically explicit environmental context of the MW. This evidence, in turn, allows examination of the military and political options available to the Mongols. Our newly obtained insight translates in an integrated model that aims to explain the sudden departure of the Mongol army from Hungary in 1242. The ‘environmental hypothesis’ that we propose, for the first time suggests that the MW may be the result of a general syndrome in which the effectiveness of nomadic armies was constrained by a short-term, regional-scale climate fluctuation.

## Results

Historical sources provide a chronological order of the Mongol invasion of and sudden withdrawal from Hungary between 1241 and 1242 CE, as well as some information on weather fluctuations and climate conditions during this time. Annually resolved, absolutely dated and spatially explicit dendroclimatological reconstructions further supplement this picture.

### Documentary evidence

**Figure 1:**
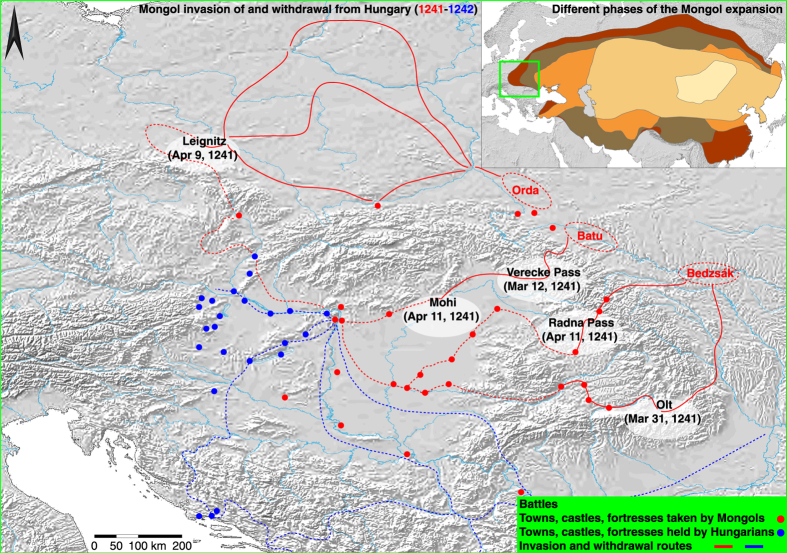
The Mongols in Eastern Europe. Spatiotemporal characteristics of the Mongol invasion of and sudden withdrawal from Hungary between 1241 and 1242 CE, with the inset referring to the different expansion phases of the Mongol empire in the 13th century. The map reflects knowledge from the authors and was created via software ArcGIS 10.1 SP1 for Desktop by Esri.

The Mongol armies entered Hungary in March 1241 (Fig. 1). The main army under Batu and Sübetei crossed the Verecke Pass in the northwest, a second army under Qadan went through the Radna Pass (in April 1241), and the third army, probably under the command of Borondai, crossed into Hungary at the river Siret/Szeret. At the same time, another army under the command of Orda and Baidar had entered Poland (Fig. 1). A few indications, such as the mention in^7^ of heat suffered by the Hungarian soldiers during the battle of Mohi (April 11), point to a warm spring in 1241 CE^17^. The Mongols do not seem to have suffered from famine in the army, and travelled across Hungary and Poland speedily. Towns and castles along their way were all conquered with relative ease. This may refer to dry soil conditions but still sufficient fodder for animals across most of the Carpathian basin.

There are no specific indications about weather in summer and fall 1241. The Hungarian provinces were partitioned among Mongol headmen^7^, and gifts and provisions were brought to them. Moreover, the Mongols did not burn the crops, ordered servants to provide shelter and fodder for their horses, and left people alive to take care of the harvest^7^. These preparations are somehow indicative of an early onset of the fall/winter in 1241. Anomalous cold conditions were accompanied by heavy snowfalls, and the Danube froze solid. Such circumstances allowed the Mongols to cross into Transdanubia and move towards current Austria and Germany. Documentation of the severity of the winter in historical sources^7^ provides the most significant direct information about climate at the time of the invasion.

In the winter of 1242, after crossing into western Hungary, the Mongols encountered more difficulties in storming fortified towns and castles than in 1241. According to (7) “when they reached the city of Székesfevehérvár that is surrounded by marshes they could not take it because the snow and ice was about to melt”. In Croatia, Qadan could not attack the city of Trogir because the flooded area separating its walls from the land was impassable on account of the depth of the mud. The army was therefore forced to withdraw^6^. The former shows that the thaw of the snow and ice may have caused large areas to become flooded and marshy, thus impeding or restricting the movement of the Mongols. The latter example suggests that the Mongols could not easily cope with flooded and muddy terrain. The captives of the Mongols were no longer given food^7^, and failed harvest caused general starvation^17^. Both the decimation and dispersal of the population caused by the Mongols^20^ and adverse climate conditions (cold and wet) may have been concurrent triggers for harvest failure, which reduced not only the survival rate of the local population, but also the sources of provisions for the Mongol army.

### Dendroclimatological evidence

**Figure 2:**
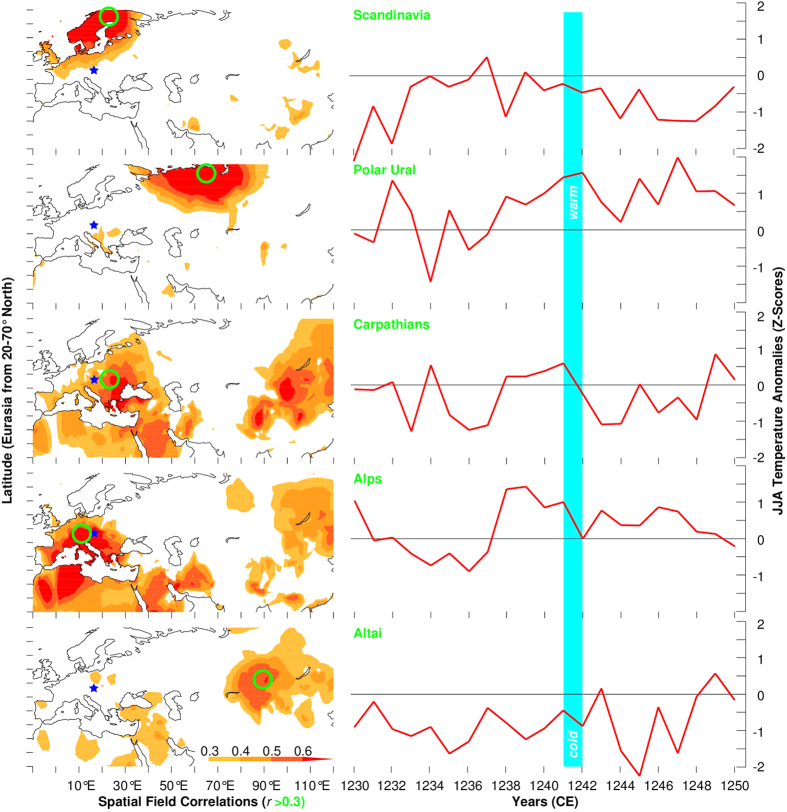
Regional temperature variability. Maps of the spatial field correlation coefficients (*r* > 0.3) of the independent summer temperature reconstructions, with the green circles denoting their locations and the blue stars referring to the MW in Hungary. All maps were created via the KNMI online server. June-August (JJA) temperature variability reconstructed from five regional tree-ring chronologies in Eurasia (~10–90°E and ~45–65°N). Time-series have been normalized (i.e. mean of zero and standard deviation of one) over the last millennium to improve visual comparison, and the vertical bar indicates temperature changes from 1241–1242 CE.

Independent tree ring-based summer temperature reconstructions from northern Scandinavia^21^, the Polar Ural^22^, the Romanian Carpathians^16^, the Austrian Alps^23^, and the Russian Altai^24^ reveal synoptic-scale differences in the estimated interannual to decadal variability (Fig. 2). While the explained variance in summer temperature over Scandinavia, the Polar Ural and the Altai is not related to Hungary and its surroundings (Fig. 2a), the records from the nearby Carpathian and Alpine arcs account for most of the summer temperature changes in central-eastern Europe. These two reconstructions reveal a plateau of four successive summers with above average temperatures from 1238–1241, followed by a sharp cooling in 1242 (Fig. 2b).

**Figure 3:**
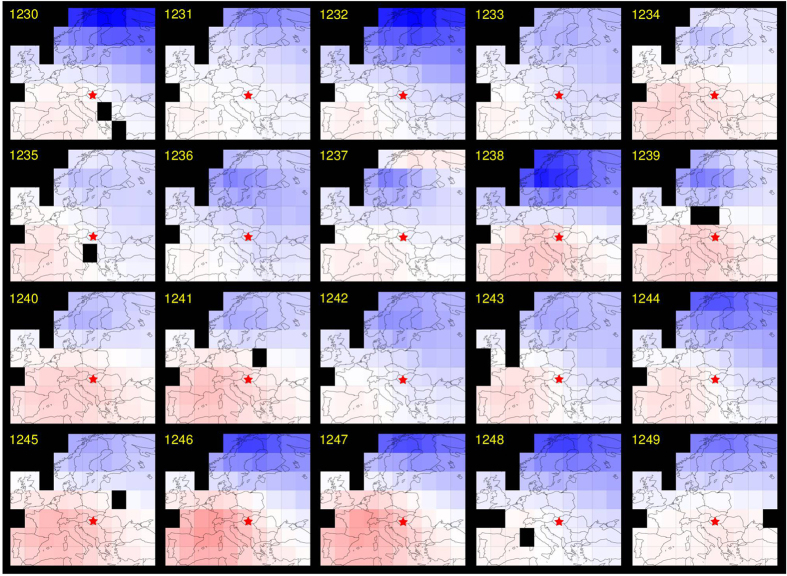
European temperature variability. Spatial field reconstruction of June-August (JJA) temperature anomalies (°C with respect to the 1961–1990 climatology) from 1230–1249 CE^16^. Maps representing a 5° × 5° land-only grid are based on a multi-proxy approach and Bayesian hierarchical modeling, with summer temperatures ranging from warm (1 °C) to cold (−2 °C). All maps were reproduced and modified from^19^.

**Figure 4:**
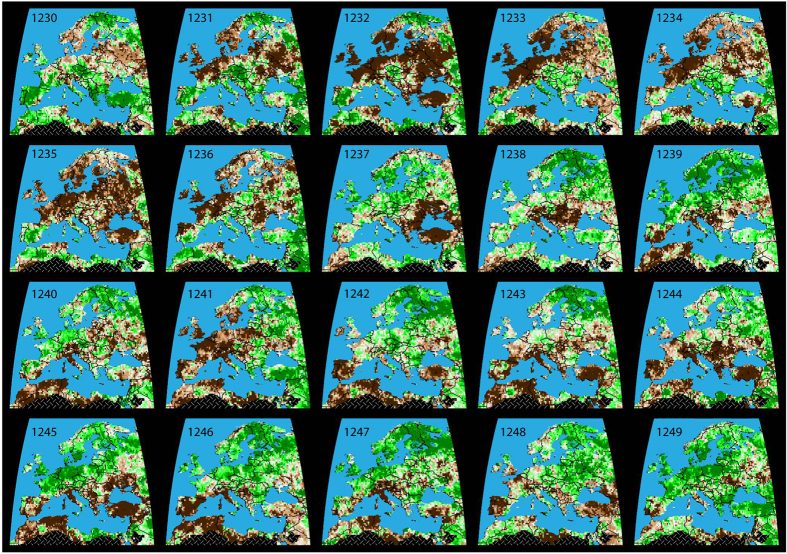
European drought variability. Spatial field reconstruction of June-August (JJA) drought anomalies (°C with respect to the 1961–1990 climatology) from 1230–1249 CE^14^. Maps representing the self-calibrated Palmer Drought Severity Indices (scPDSI) at a spatial resolution of one-half-degree longitude by latitude are based on the OWDA, which reflects regional soil moisture conditions between dryness (−6; brown) and wetness (6; green). All maps were reproduced and modified from^18^.

Spatial field reconstructions of European summer temperatures^19^ between 1230 and 1250 indicate overall cooler summers over northern Scandinavia but relative warm conditions over the Iberian Peninsula, relative to a long-term mean (Fig. 3). An uninterrupted cluster of above average temperatures over Hungary and its surroundings was found between 1238 and 1241. Colder summers, however, occurred from 1242–1244. Spatial field reconstructions of European summer drought^18^ between 1230 and 1250 reveal distinct regional differences in hydroclimatic variability (Fig. 4). An overall heterogeneous picture implies dryness over much of northwestern Europe, including Scandinavia and the Baltic from 1232–1235. Southern and southeastern Europe, in contrast, more often experienced dry summer conditions between 1237 and 1248. Extensive pluvials in 1238 and 1239 were mainly restricted to Scandinavia but frequently also expanded over the central and eastern part of the continent after 1242. Most of Europe from the Iberian Peninsula in the southwest to the Baltic in the northeast exhibited anomalous dry conditions in 1241. The exceptional wetness of 1242 was, however, spatially restricted to southern Poland, most of the Czech Republic, western Slovakia, northwestern Hungary and eastern Austria. While above average wetness in this region continued until 1246, dryness characterized its southern and eastern surroundings at that time.

## Discussion

Our case-study of the MW from Hungary illustrates the incidence of even small climate fluctuations upon a major historical event. In this respect it is necessary, first, to discuss the progress of the invasion in military terms and the measures taken by the Mongols to consolidate their occupation. Secondly, we consider the ways in which climate probably impacted the effectiveness of the Mongol army, based on specific characteristics and limitations in a certain region (which might have been different in other regions, e.g., drought versus temperature limited environments). Finally, we argue how environmental factors may have entered the decision-making process of the Mongol leadership (at a specific location and within a short period).

During their invasion in 1241, the Mongols moved swiftly across the country and were initially successful in taking various fortresses, destroying any resistance they encountered. The population bore the brunt of the subsequent occupation, and villages were left empty as the people were killed or fled into the forests and hills. In some cases they were captured, enslaved, and drafted into the Mongol army. Significant elements to consider in this scenario are: First, no serious armed resistance hindered the Mongol occupation after the battle of Mohi. Second, plundering was extensive, and third, the Mongols set up a rudimentary administrative system in which several villages were placed under the control of Mongol leaders^7^, presumably for fiscal and civil order purposes. We should also take note of the fact that the Mongols, as per Roger’s testimony^7^, did not burn the fields, and took care not to harm the harvest or fruit-bearing trees. Attention to productivity indicates that the Mongols did not intend to turn agricultural land into pasture, which would have presumably been the case had grass been insufficient.

The Mongols did not cross the Danube into the western part of Hungary until January or February of 1242, when several sources attest that the river froze, allowing the army to cross and engage in the devastation of western Hungary. Moreover, abundant snowfalls are recorded for that time. The Mongols who reached Croatia in pursuit of King Béla IV also found swampy conditions and eventually withdrew without being able to inflict much damage on the cities. In contrast to the surrounding hilly terrain where Luvisols dominate, much of the Hungarian plain is covered by relatively young soils without distinct profile development, so-called Cambisols^25^. Dark Chernozems are widespread throughout the Carpathian basin and represent the most fertile soil type. River valleys that have developed on stratified sediments are characterized by so-called Fluvisols, whereas Arenosols have developed on windblown sands deposited after the end of the last ice age. Most of the lower elevation soil types in Hungary are particularly prone to stagnant moisture and ponding. These areas may turn quickly into marshy terrain when increased snowmelt and rainfall coincide at the seasonal transition from winter to spring. Soil wetness, in turn, not only delays the onset of the vegetation period but also reduces the overall productivity of the extensive agricultural and natural grassland habitats in Hungary.

The military situation in the late winter and spring of 1242 therefore seems to have changed. The Mongols encountered considerable difficulties in taking fortified castles and citadels, for instance. While stone fortresses offered greater resistance, the Mongol failure is explicitly attributed in the sources to swampy terrain. It is possible that these conditions made it much more difficult to operate siege engines and to keep a cavalry force in the surrounding areas. Moreover, the early spring thaw may have severely reduced the amount of grazing land, with horses already weakened by the winter, and thus limited the movements of the Mongols to areas with available pasture. The reduced population, abandonment of fields and devastation of large parts of the country must have also reduced the Mongols’ ability to procure victuals. The combined effects of the war and a less favorable climate may have also caused the failure of the harvest of 1242 and the ensuing ‘great famine’ in Hungary^17^. It should be further noted that military operations of inner Asian nomads, to which the Mongols were no exception, were normally executed in autumn and continued through the winter, while the spring and summer were seasons in which they were at their weakest and most vulnerable. Military seasonality is part and parcel with the pastoral economy upon which the Mongols depended, and with the management of its resources, primarily horses. According to contemporary sources, the Mongols did not provide forage for the horses but allowed them to graze freely in the grassland^26^. This indicates an obvious vulnerability in case no sufficient grass was available or in easy reach.

It is therefore under conditions of (i) reduced mobility and military effectiveness; (ii) reduced fodder for the horses; and (iii) reduced victuals for the army, which in the late spring of 1242 the Mongols left Hungary. The main Mongol army withdrew towards east following the southern course of the Danube (Fig. 1), thus crossing Serbia into Bulgaria, where they obtained the submission of the king Kaliman I at Tarnovo, before crossing Wallachia and Moldavia and returning to the steppes in the lower Volga region. A secondary army under Qadan that had travelled to Dalmatia in pursuit of King Béla followed the same route, joining the main army. Some minor contingent, such as the troops that had captured Roger may have proceeded to return through Transylvania. It is difficult to say why Batu chose to return by a southern route, but it is possible that the army moved to overall dryer and higher ground along the Carpathian foothills to avoid marshy conditions. In this respect, we may recall that years later the Mongol khan Hülegü mentioned in a letter that it is the Mongol habit, when the summer is hot, to move to the fresh and snowy mountains, and because during his campaign in Syria the grass and food had been for the most part used up, some troops were sent to the mountains of Greater Armenia^15^. The southern withdrawal route may therefore have been suggested by the drier conditions of a higher altitude, while the Hungarian plains were marshy and devastated by famine.

**Figure 5:**
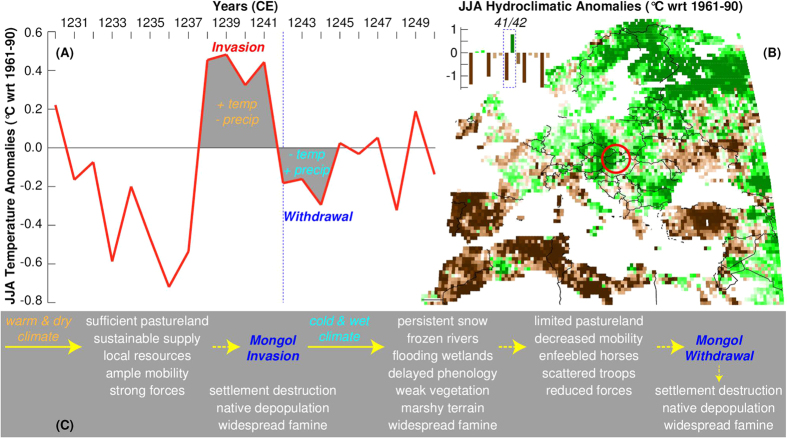
The Mongol withdrawal. (**A**) June-August (JJA) temperature anomalies (°C with respect to the 1961–1990 climatology) averaged from the two independent tree ring-based summer temperature reconstructions from the Austrian Alps^12^ and Romanian Carpathians^11^. (**B**) Map of the OWDA showing June-August (JJA) soil moisture anomalies for 1242 CE. (**C**) The putative interplay of various environmental factors, resulting in more or less favorable conditions that either increase or decrease the number of options for the Mongol army during their successful invasion of and sudden withdrawal from Hungary, respectively. The map was reproduced and modified from^18^.

In our ‘environmental hypothesis’ (Fig. 5), we join paleoclimatic with historical information to show the possible impact of even small climatic fluctuations and time-specific environmental conditions on a set of historical circumstances. In so doing, we demonstrate that relatively modest changes can have a decisive impact. In this manner, for instance, we replace the static relationship between environment and human response – based on a constant notion of carrying capacity of the land – with a dynamic concept that estimates actual year-by-year conditions, and thus allows us to perceive the precise constraints under which decisions were taken. The data presented here support the view that the climatic conditions that occurred in Hungary between 1241 and 1242 had a considerable impact on the productivity of the land as well as on the suitability of the terrain for military operations of the type performed by the Mongols (see also details above), relying on high mobility and swift storming of fortified places. In the winter of 1241–42 lower temperatures initially favored the movement of the Mongols by allowing them to overcome frozen rivers, but later the thawing of the snow and ice made the land wet and marshy. The failure of the Mongols to take fortified castles may be at least partially attributed to such unfavorable conditions. The alternating environmental circumstances probably made it difficult for the horses to find grass in sufficient quantity and for commanders to concentrate their forces in sufficient numbers to practice their usual tactics (Fig. 5). Moreover, the Mongols no longer fed their prisoners other than meager scraps of food^7^, which indicates that the great famine that was soon to hit Hungary could be foreseen, placing an additional obstacle on the Mongols’ occupation. Our ‘environmental hypothesis’, therefore, argues that small climatic changes from 1241–42 were though sufficiently extensive to alter the conditions under which the Mongols first invaded Hungary. These changes would not allow them to function effectively as an occupation army, thus forcing them to withdraw.

The Mongol conquest operated according to multiple models and conditions specific of the several places and objectives. The initial campaign in Khurasan lasted several years and resulted in recruitment of local administrators and elites, which provided a basis for later campaigns and consolidation of Mongol rule in that area. Different phases of the conquest corresponded to different, sometimes limited objectives. The early campaigns in northern China mostly aimed to impose tributes and other demands, evolving only later into a model of territorial conquest. Therefore, we can see that the Mongol empire changed considerably from the tributary demands imposed by Chinggis Khan, to the phase of actual conquest that took place under Ögödei, and later imperial expansion under Möngke. The campaign in Hungary belongs to the second phase, under the khanship of Ögödei, which focused more closely on territorial occupation and can regarded more similar, and indeed an extension of the Russian campaign. This was not accomplished in multiple waves, but in a single multi-year campaign aimed to obtain the quick surrender of the local towns and imposition of tributes, while Mongols settled in areas that were ecologically suitable to their economy, lifestyle, and military needs (the Volga basin). The Hungarian branch of the campaign was one of the western campaigns under Batu. That the Mongols stayed in southern Russia and did not seriously attempt to invade eastern Europe again (with the exception of a short-lived invasion of Poland in 1259) has not been so far an object of historical inquiry. However, this paper raises the possibility that the vulnerability of the Hungarian plains to even relatively short-term climate events made it obvious that the region was unsuitable for military occupation by a large army relaying mostly on horses. It is worth noting that the Hungarian river system was prone to flooding and to creating marshlands, and only much later it became drier, thanks to drainage work undertaken by the Hapsburgs in the 19th century^27^,^28^. Explanations for the Mongol strategies have to take into consideration local conditions as well as the specific objectives of different phases of the conquest. We may also recall that the absence of a repeated Mongol invasion can be compared with the two failed invasions of Japan in the late-13th century, which were not followed by further attempts. Our paper shows that a possible reason why the Mongols who occupied Russia under Batu and his successors did not make further attempts to expand westward may have depended on the realization that local conditions would not have supported a prolonged occupation. While the reasons why the conquest of the West halted in southern Russia have to remain speculative in the absence of proper documentation, we should consider environmental conditions on a par with political ones, such as the civil war that engulfed the Golden Horde and the Il-Khanate in following decades, and cultural ones, such as the Turkicization and Islamization of the Golden Horde, which transformed the Mongol leadership. Moreover, economic changes brought about by the development of trade also caused a change of attitude towards the West, whereby Venetian and Genoese trading towns were established on the Black Sea, in the territory under Mongol rule in the early-14th century.

We finally conclude that the advantages deriving from a joint analysis of historical and paleoclimatological data are especially relevant to the reconstruction of a more articulate and richer context, rather than to the construction of causal chains. Further insight should be produced, if available, from archaeological and other disciplines, including both natural sciences and the humanities. It would be, for instance, extremely important to examine the remains of the castles and fortifications that the Mongols stormed or failed to take. Studies on historical changes in the hydrology of Hungary would also provide relevant information about the geography and ecology of Hungary in the 13th century. Moreover, the extent to which war, devastation and famine impacted the local population may also surface through epidemiological studies, coupled with isotopic analysis to investigate dietary changes. Finally, the extraction of aDNA would inform us of the extent to which Mongol and Cuman invasions altered the genetic makeup of Hungary and its surrounding regions. While caution must be exercised when extrapolating complex mechanisms of human-environment interaction over space and time^24^, the MW constitutes an excellent, though perhaps still preliminary, case-study to explore the role that relatively minor environmental factors may have played on major socio-cultural, political and economic phenomena. The example of the MW also stresses the benefits of creating a science-informed and data-rich context for seeking explanations of historical events^24^.

## Methods

Climate and weather-related information from historical archives related to Hungary in 1241 and 1242 were revisited^17^. The main sources are the account of the Mongol invasion by Rogerius^7^, the Italian archdeacon of Nagyávarad^17^. Additional important sources are the chronicle by Thomas of Spalato^6^, which has been extensively studied by Sweeney^29^. Kiss^17^ has also mined several collections of medieval documents and chronicles, such as the *Codex diplomaticus Hungariae ecclesiasticus et civilis*, compiled by György Fejür^17^,^30^, the *Anonymi Chronicon Austriacarum* and the *Continuatio Sancrucensis*. Additional information comes from Persian sources^31^. The information on the routes taken by the Mongols and related geographical information has been thoroughly documented by Tátar based on medieval document in collections such as the *Catalogus fontium historiae Hungaricae aevo ducum et regum ex stirpe Arpad descendentium ab anno Christi DCCC usque ad annum MCCCI. I-III*^30^.

Five independent tree-ring proxy records from northern Scandinavia^21^, the Polar Ural^22^, the Romanian Carpathians^16^, the Austrian Alps^23^, and the Russian Altai^24^ were used to reconstruct year-to-year and longer-term changes in regional to synoptic-scale summer (June-August; JJA) temperatures between 1230 and 1250 CE. Newly developed spatial field reconstructions of European summer temperature^19^ and drought^18^ were used to generate spatially explicit maps of the climate conditions during this time. Low-frequency estimates of total solar irradiance changes^32^ and precisely dated volcanic eruptions^33^ suggest that the MW occurred during a period of increased solar and reduced volcanic activity. Independent paleoclimatic evidence from different proxy archives, including Alpine glacier fluctuations^34^,^35^ and Carpathian peat profiles^36^ provides further insight into longer-term temperature and hydroclimatic changes, roughly after medieval times and before the onset of the so-called Little Ice Age type events^37^.

## Additional Information

**How to cite this article**: Büntgen, U. and Di Cosmo, N. Climatic and environmental aspects of the Mongol withdrawal from Hungary in 1242 CE. *Sci. Rep.* **6**, 25606; doi: 10.1038/srep25606 (2016).
